# A new technique for Asian nasal tip shaping: "twin tower" folding ear cartilage transplantation

**DOI:** 10.1080/23320885.2022.2123807

**Published:** 2022-09-14

**Authors:** Long Zhang, Jiang-wen Wang, Jun Ding, Xi Zhang, Xi-mei Wang, Zhan-zhao Zhang, Run-ze Yu

**Affiliations:** a Department of Plastic Surgery, Medical Cosmetology Hospital of Yestar Hangzhou, Hangzhou, China; b Linping Campus, The Second Affiliated Hospital of Zhejiang University School of Medicine, Hangzhou, China; c Department of Plastic Surgery, The First Affiliated Hospital of Zhengzhou University, Henan, China; d Aesthetic Surgery Clinic of Yestar Jiaxing, Jiaxing, China

**Keywords:** Auricular cartilage, septal extension graft, three-dimensional anthropometric analysis, rhinoplasty

## Abstract

Rhinoplasty focuses on the establishment of the structural support of nasal cartilage and the shaping of the nasal tip. The purpose of this study was to explore the application of "double tower" folding ear cartilage transplantation for nasal tip shaping in rhinoplasty.

## Background

The nose is located in the middle of the face and is a key part of facial aesthetics. In Asian people, the face is flat, and the skin and soft tissues of the nose are generally thick, resulting in a round and blunt appearance of the nose [1]. The nasal septum is short, and the lower lateral cartilage is narrow and small; in particular, the medial foot of the lower lateral cartilage is small, forming a low, flat appearance [2]. Therefore, heightening and prolonging the Asian nose plastic will elevate the nose relative to the contour of the face, and the apparent depression of the face will result in obvious improvement [3].

Rhinoplasty is a common, yet relatively complex, operation in cosmetic surgery, and the focus is on the establishment of structural support for the nasal cartilage and shaping of the nasal tip [4]. In recent years, many Western Dallas rhinoplasty techniques and concepts have been promoted in China, but for Orientals, most of their noses are not three-dimensional enough. Therefore, it is necessary to carry out rhinoplasty surgery according to the characteristics of Orientals. Most of the surgical plans we take are mainly to raise the bridge of the nose and the tip of the nose [5]. Autologous cartilage is the material of choice in nose tip shaping, including ear cartilage, septal cartilage, and costal cartilage, but each has its own advantages and disadvantages. In clinical practice, we found that compared with the nasal septal cartilage or costal cartilage, the application of ear cartilage in nose tip shaping can also achieve the same effect of elevating the nose tip and lengthening the length of the nose tip [5].

We constructed a “double tower” nasal stent by folding and suturing the ear cartilage in half, which could increase the tip of the nose, improve the retraction of the nasal columellar and short noses, increase the supporting force of the ear cartilage, and maintain long-term stability after rhinoplasty. From May 2018 to June 2019, we conducted clinical observations in 38 patients undergoing this method and achieved good results, which are reported as follows.

## Methods

### Patient information

A total of 38 patients who underwent double-tower folding ear cartilage tip plasty at Hangzhou Yixing Medical Cosmetic Hospital from May 2018 to June 2019 were included in this report. There were 3 male patients and 35 female patients aged 18–35 years old. We eliminated heavy saddle noses, severe short noses and contracture in patients, totaling 22 cases of primary surgery and 16 cases of secondary surgery. A second surgery was mainly required for a simple prosthetic nose job or when nasal tissue damage was not serious, after which the comprehensive edges, ear cartilage and nasal septum cartilages remained relatively intact in all 38 cases.

Inclusion criteria: patients with congenital saddle nose deformity, round, blunt and flat nose tip or too short nose length; patients with poor shape after injection rhinoplasty and prosthetic rhinoplasty seeking secondary repair. Exclusion criteria: including but not limited to coagulation disorders, heart disease, pregnant women; severe short nose (The length from the base of the normal nose to the tip of the nose is about one-third of the full face. If the length of the nose is less than one-third of the full face, it is a short nose. If the length of the nose is less than 5 mm or more than the normal value, the nose is rounded and blunt, the nostrils are exposed, and the root of the nose and the dorsum of the nose is severely flattened.) and contracture nose with severe tissue damage; severe deviated septal cartilage; high surgical expectations or pursuit of exaggerated effects.

### Surgical methods

**Figure 1.**
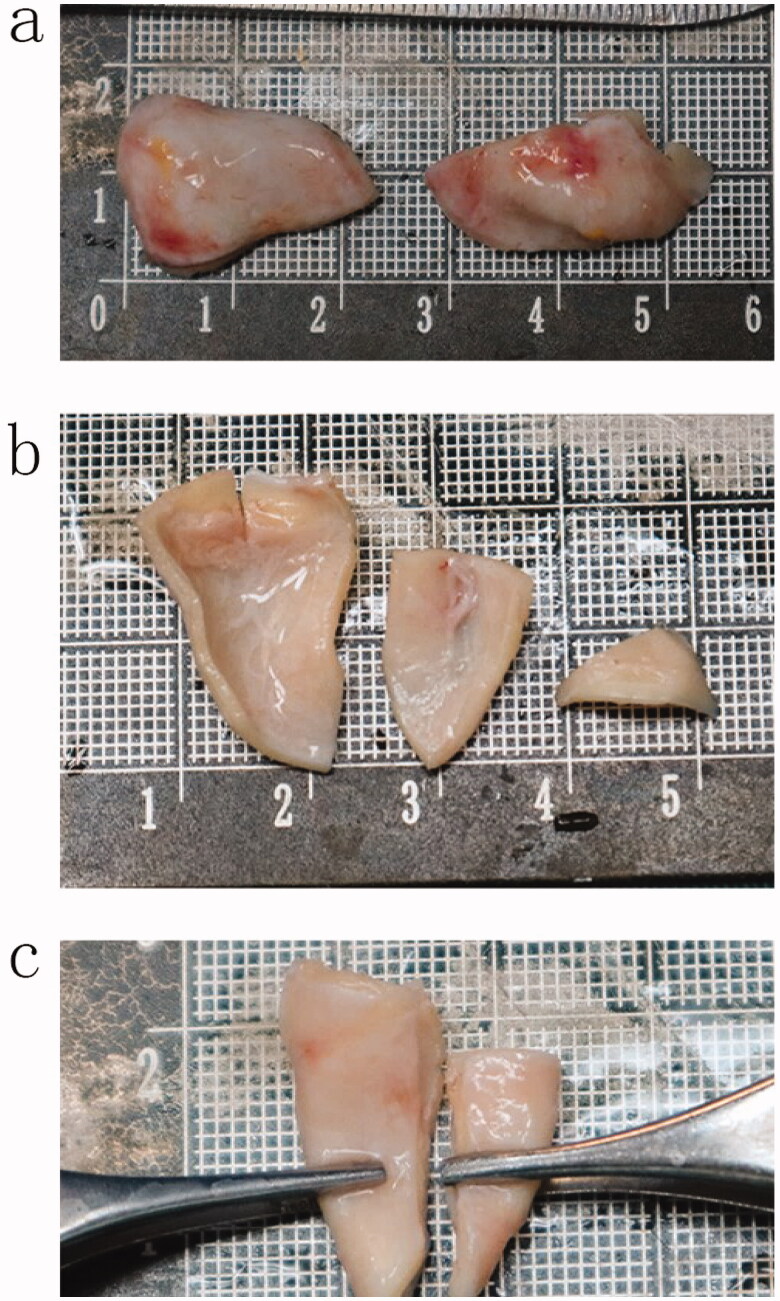
(a) Two pieces of ear cartilage removed; (b) The ear cartilage has been processed; (c) Two pieces of ear cartilage with folding function.

The auricular cartilage was obtained through a retroauricular incision, and the auricular cartilage from the auricular cavity was cut from both sides of the ear of the patient. When the auricular cartilage was cut, it measured 2.5–2.8 cm long and 1.2–1.5 cm wide (Video 1). When cutting the auricular cartilage, we generally acquired a graft approximately 1.5–2 cm wide and 2.5 cm long (Video 2). These numbers are the average of the included patients. After the ear cartilage was removed, there was a small amount of fascial muscle tissue on the back, which could be removed and used to cushion the nose. The ear cartilage was removed to appropriately trim the convex edge, cutting as little as possible. The cartilage of the removed auricular cartilage was cut at the thin tip with a length of 5 mm and a width of 8 mm and was used as a cap graft or shield graft at the tip of the nose (Video 3; Figure 1).

**Figure 2.**
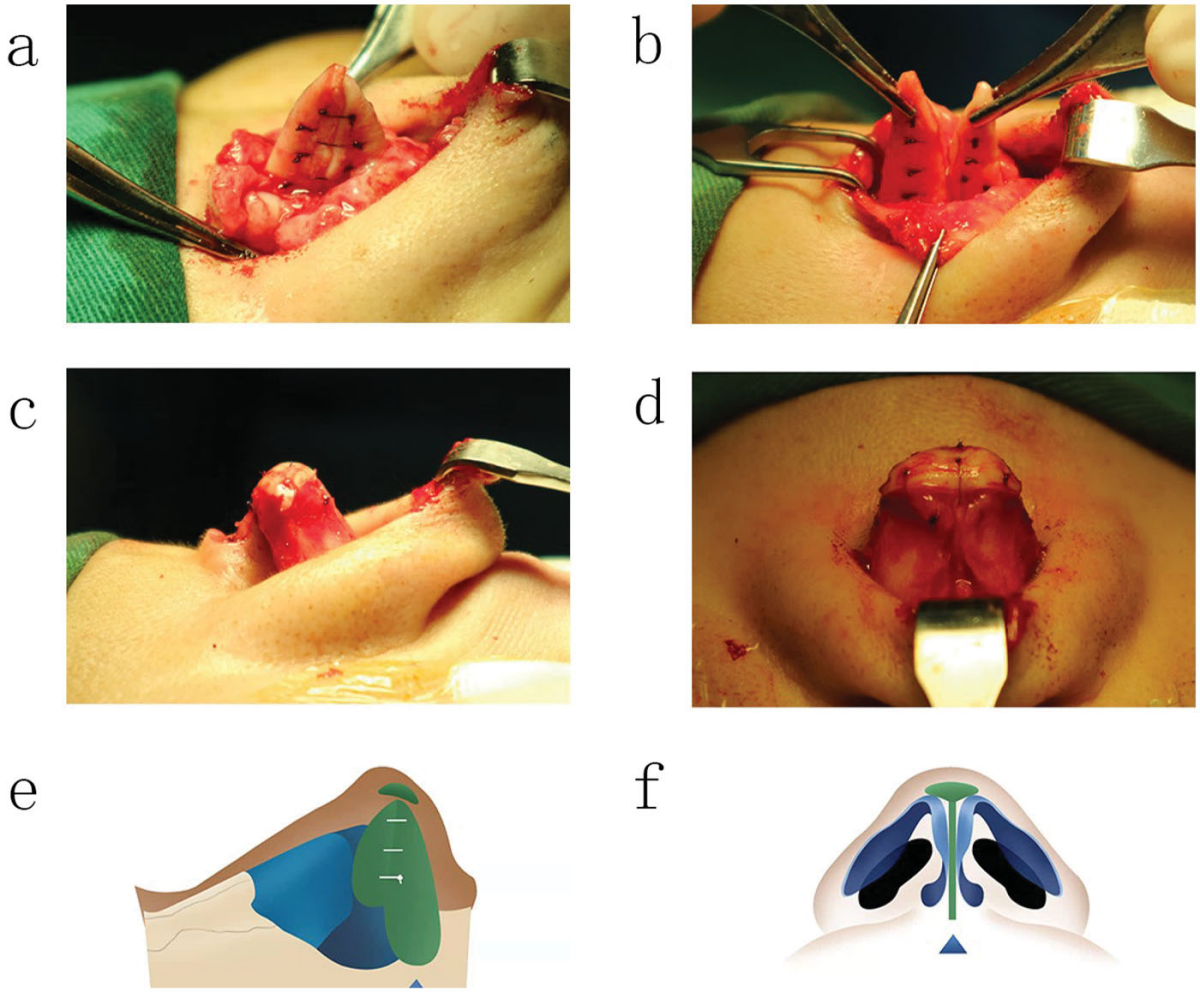
(a) Double tower folded ear cartilage fixed to the nasal septum; (b) folded ear cartilage fixed to the nasal septum; (c) the cap ear cartilage is sutured at the tip of the nose (Lateral view); (d) the cap ear cartilage is sutured at the tip of the nose (anteroposterior view); (e) diagram of the surgical procedure (Lateral view); (f) diagram of the surgical procedure (anteroposterior view).

We cut the skin of the columella through an inverted V incision, cut the skin to the fornix along both sides of the columella and at 3 mm from the edge of the columella, cut the skin along the caudal edge of the alar cartilage to the vestibule of the nasal cavity, and peeled off the lower lateral cartilage on the perichondrium. The flap was pulled upward to fully expose the bilateral alar cartilage, and the cartilage links and ligaments in the scroll area were released. There was no obvious resistance when the alar cartilage dome was lifted, which was conducive to lifting or pulling down the alar cartilage. We separated the alar cartilaginous vault and medial ligament between organizations, separated the septal membranes, exposed the nasal septal cartilage under the perichondrium, removed approximately 5–8 mm wide strips of septal cartilage under the perichondrium on both sides and separated the deep muscle fascia tissue around the nasal spine and cartilage to perform the reconstruction. The removed cartilage was folded in half toward the cephalic end and stuck on the septum, which was sutured and fixed on the caudal end of the septum. According to the thickness and length of the septal cartilage, the amount of overlap between the ear cartilage and nasal septal cartilage could be determined. At this point, an intact ear cartilage scaffold was formed (Video 4; Figure 2). The sutures used for the fixation of the ear cartilage are 5.0 nylon thread and 5.0 PDS thread. which was equivalent to lengthening and elevating the nasal septum. Finally, the prepared otoconia cartilage was sutured and fixed at the tip of the nose as a cap graft or shield graft, and the fascia muscle tissue on the back of the removed otoconia cartilage was covered on the shield graft or cap graft so that the tip of the nose was basically formed (Video 5; Figure 2). Based on the new height of the original back from the tip height and strip dorsum lacuna, the placement of bulk or silicone prostheses was sized to observe the nose shape and was adjusted again after the prosthesis was placed. Small incisions were made to achieve nasal shape satisfaction. Then the two nostrils were covered with a petroleum jelly gauze strip, the nasal tip and nasal back were covered with adhesive plaster, and the nasal plastic plate fixation was repositioned.

### Patient satisfaction assessment and anthropometric analysis

We conducted postoperative telephone follow-up of patients through questionnaire a. The photos were compared before and 6–12 months after the operation and were evaluated by doctors, patients and a third party: if the three parties were satisfied, it was regarded as significantly effective; if two were satisfied with it, it was effective; if one was satisfied with it, it was average; and if none were satisfied, it was graded as poor. Efficacy satisfaction = (significant effective + effective)/total number of cases × 100% [6].

In addition, we also analyzed the preoperative and postoperative differences by measuring four linear nasal datasets and three angle datasets. Linear data were measured as follows: nasal length (straight-line distance between root point and tip point), nasal height (straight-line distance between root point and subnasal point), nasal width (distance between outermost points on both sides of alar), and nasal depth (straight-line distance between the subnasal point and tip point). Nasolabial angle (angle between the columella line and the line connecting the subnasal point and the upper lip point), nasofrontal angle (angle between the nasal dorsum line and the oblique plane from the forehead to the nasion), nasal tip angle (angle between the nasal dorsum line and columella line) [7].

### Statistical analysis

Data were analyzed using Prism software, version 8.0 (GraphPad Software, San Diego, CA, USA). We analyzed and compared the differences between preoperative and postoperative nasal parameters using the paired *t* test. All data are expressed as the mean ± standard deviation. *p* < 0.05 indicated statistical significance.

## Results

### Patient satisfaction

**Figure 3.**
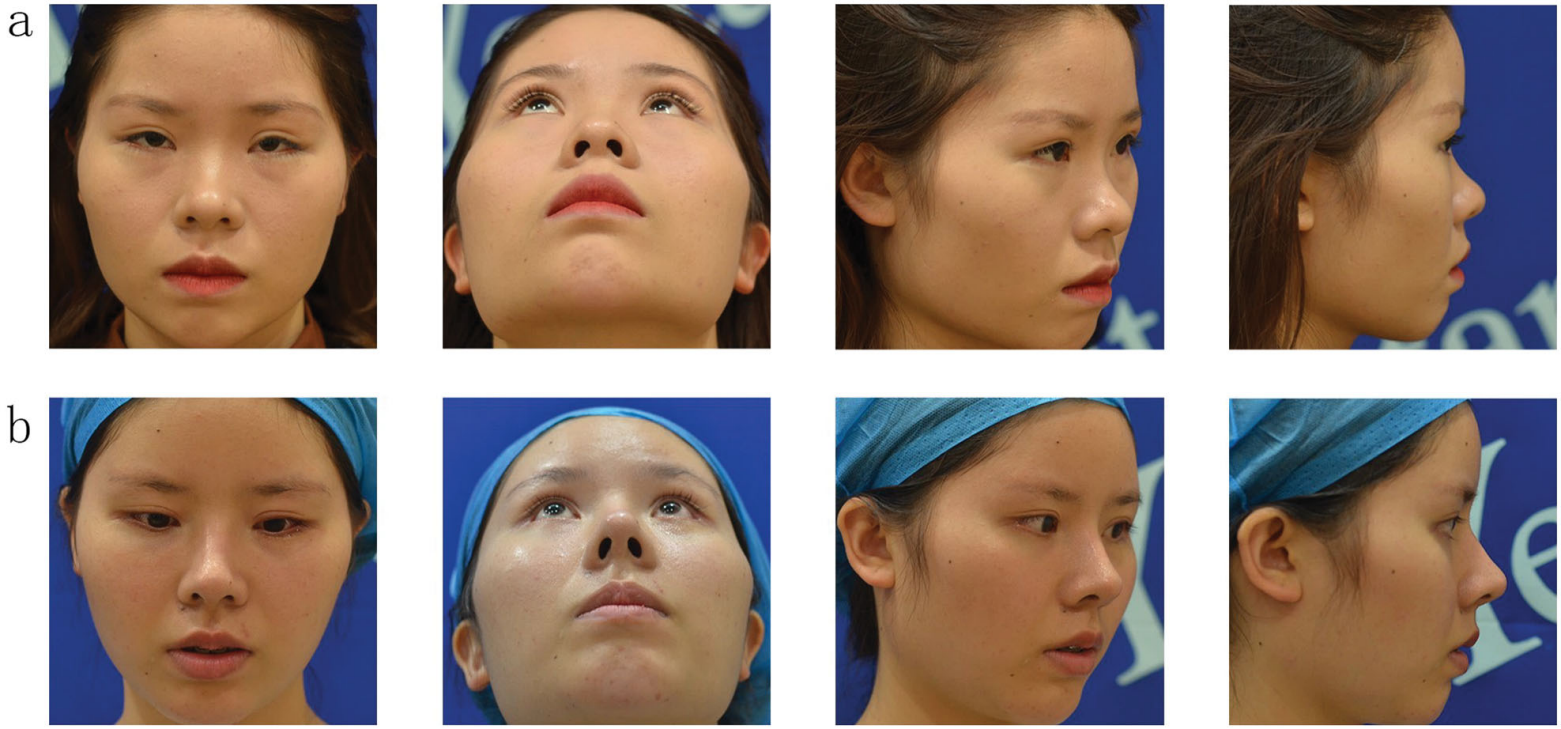
A 24-year-old Asian woman underwent twin tower folding ear cartilage rhinoplasty. Preoperative (a) and postoperative (b) facial profiles are shown.

**Figure 4.**
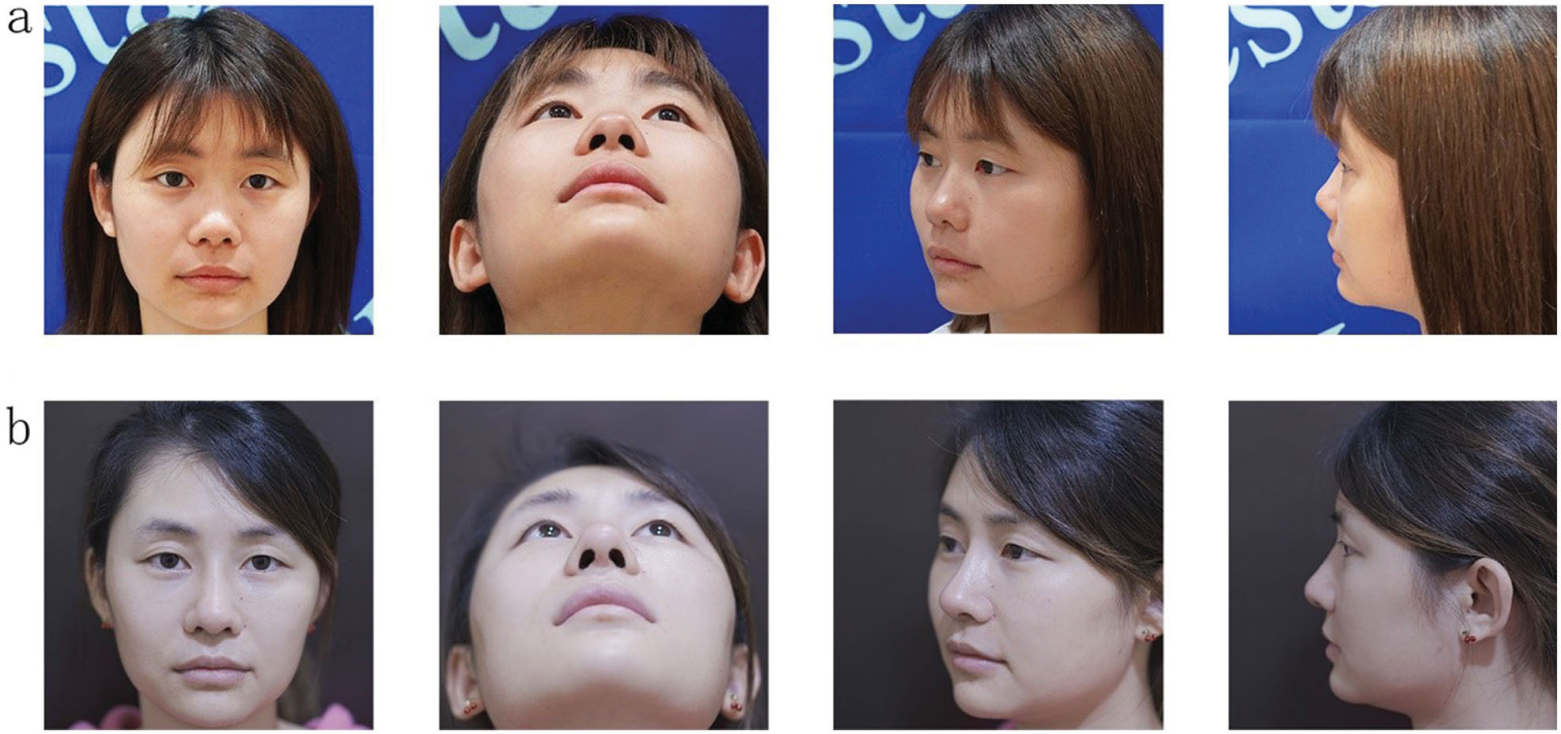
A 28-year-old Asian woman underwent twin tower folding ear cartilage rhinoplasty. Preoperative (a) and postoperative (b) facial profiles are shown.

Thirty-eight patients in this group were followed up for 6–12 months, and all nasal incisions healed well without hematoma or infection. Among the patients, 2 refused to receive implant prostheses in the nose before surgery but felt that the nasal root and nasal dorsum were slightly lower after surgery and then had prostheses implanted in the nasal dorsum again. The postoperative recovery was good, and the patients were satisfied. For 2 patients, the absorption of the ear cartilage transplant became soft half a year after surgery, and the nose tip was slightly reduced. The patient required the nose tip to be increased, and then the ear cartilage was removed as a cap graft pad at the nose tip. The nose tip was increased, the postoperative recovery was good, and the patient was satisfied. The other patients had a full nasal shape, smooth side, smooth arc, symmetrical bilateral nostril, prolonged and elevated nasal tip, the morphology was significantly improved, and the prosthesis was not active, deflected or twisted. There were 3 cases of premature removal of the ear compression dressing and consisting of cotton balls, after surgery, resulting in a small hematoma, which caused the patients to return to the hospital for a puncture to extract part of the congestion and reapplication of the compression dressing. The remaining small amount of hematoma was spontaneously absorbed, the wounds healed normally without obvious ear deformation, and the auricular cavity became slightly shallow. The other 35 cases healed normally without hematomas, no obvious incision scars, no obvious shallow auricular cavities and no obvious changes in auricle shape. Among the 38 cases, 26 cases were significantly effective, 10 cases were effective, 2 cases were general, and the satisfaction rate of efficacy was 94.74% (Figures 3 and 4).

### Comparison of preoperative and postoperative nasal measurements

The measurement and analysis results are shown in Table 1. The mean preoperative nasal length of the patient was 40.64 mm ± 4.81 mm. The mean postoperative nasal length was 45.89 mm ± 5.21 mm (*p* < 0.05). The mean preoperative nasal width was 37.86 mm ± 2.97 mm. The mean postoperative nasal width was 35.53 mm ± 2.44 mm (*p* < 0.05). The mean preoperative nasal height was 50.91 mm ± 5.05 mm. The mean postoperative nasal length was 54.76 mm ± 5.26 mm (*p* < 0.05). The mean preoperative nasal depth was 17.68 mm ± 1.01 mm. The mean postoperative nasal depth was 18.89 mm ± 1.52 mm (*p* < 0.05). The mean preperative naso-frontal angle was 138.36°±7.25°. The mean naso-frontal angle was 144.38°±6.99° (*p* < 0.05). Similarly, the mean nasolabial angle was 101.66°±5.17° compared with 95.93°±6.12° before surgery (*p* < 0.05). However, the mean nasal tip angle was 87.61°±5.81° before surgery. The mean nasal tip angle was 86.89°±5.21° after surgery (*p* > 0.05), and there was no significant difference. All data have been tested for normality and are normally distributed.

**Table 1. The preoperative and postoperative measurement and analysis results.** 

	Preoperative values	Postoperative values	*p*	*t*
Nasal length (mm)	40.64 ± 4.81	45.89 ± 5.21	<0.0001	4.564
Nasal Height (mm)	50.91 ± 5.05	54.76 ± 5.26	0.0017	3.255
Nasal width (mm)	37.86 ± 2.97	35.53 ± 2.44	0.0004	3.737
Nasal depth (mm)	17.68 ± 1.01	18.89 ± 1.52	0.0001	4.069
Nasolabial angle (°)	95.93 ± 6.12	101.66 ± 5.17	<0.0001	4.409
Nasofrontal angle (°)	138.36 ± 7.25	144.38 ± 6.99	0.0004	3.685
Nasal tip angle (°)	87.61 ± 5.81	86.89 ± 5.21	0.5713	0.5687

## Discussion

It is more common for Asian people to have flat faces and shorter, lower and flat noses than for Caucasian people to have them [8]. This situation is related to the development of bone and cartilage in our noses, as well as the development of the skin and mucous and soft tissues [8]. Asian people’s skin and subcutaneous tissue on their noses are generally thicker, and the growth of cartilage is generally relatively weak. The procedure described here aims to increase the height and length of the nose, hence rhinoplasty, in addition to the separation of the dorsal skin soft tissue, increases its compliance and ductility. Likewise, the alar cartilage and lateral crura and the release of the scroll area, increase the alar cartilage and stretch the mucosal lining. It is very important to construct cartilage scaffolds and shape the nasal tips [9]. With the development of rhinoplasty in China, there are a variety of methods for the construction of cartilage scaffoldings and nasal tip formation, and the materials are also more extensive. The commonly used autologous cartilage for the construction of cartilage scaffoldings and nasal tip formation comes mainly from the ear, costal and nasal septum cartilage [10,11].

The septal cartilage is harder and stronger than the ear cartilage, and the cut cartilage is flat without bending [12]. Therefore, it is mostly used for nasal columella support grafts and nasal septum extension grafts. However, the disadvantages are: the nasal septal cartilage in Asians is relatively weak, with an average thickness of 0.2 cm, and most of them are accompanied by different degrees of nasal septum deviation; the supporting force of the nasal septal cartilage is insufficient, resulting in inconspicuous nasal tip performance points; the nasal septal cartilage is relatively insufficient, and can only be The lateral fixation is on the caudal end of the nasal septal cartilage, and ear cartilage is often required to strengthen the stability [13–15]. Rib cartilage has many advantages and a wide range of materials, which is suitable for a nose with poor foundation and nose repair. However, rib cartilage rhinoplasty is more traumatic, has many complications, and often requires hospitalization, so many patients do not accept this method [16]. Ear cartilage belongs to elastic cartilage, which has good elasticity, soft texture, strong plasticity and sculpting, and is more convenient to obtain, which can meet various needs in rhinoplasty [17].

The nasal scaffold constructed by ear cartilage is also based on the nasal septum cartilage and maxillary nasal spine. A piece of the ear cartilage is folded cephalad and then stuck on the nasal septum, equivalent to the extended graft of the nasal septum and the support graft of the nasal columella. According to the hardness and length of the nasal septum cartilage, the ear cartilage stent can determine how much the ear cartilage overlaps with the nasal septum cartilage to extend the nasal septum and help shrink a short nose or nasal columella. It can also move back and forth to permit fixing onto the middle or posterior horn of the caudal margin of the nasal septum according to need, and reasonably raise the height of the nasal tip. The two auricular cartilages were folded in half and fixed on the caudal margin of the nasal septum to form two isolated "tower"-shaped cartilages. After suturing the two together, a cartilage scaffold resembling a triangle was formed. We use the prosthesis combined with ear cartilage transplantation instead of autologous tissue. The reason is that the amount of autologous ear cartilage taken out is not enough. If only autologous tissue is used, it is not enough to completely adjust the height of the nasal dorsum.

The principle of the "moment of inertia" is used to cause the folded ear cartilage to significantly increase the effects of compression and torsion resistance, thus maintaining long-term stability after rhinoplasty [18,19]. The moment of inertia of the ear cartilage folded in half was more than 100 times that of the ear cartilage not folded in half. The moment of inertia of a material is a mechanical index reflecting its anti-bending ability. Therefore, the increase in the moment of inertia of the auricular cartilage after folding it in half indicates that the auricular cartilage scaffold after folding cartilage in half has the greater anti-bending ability, providing strong objective support for our method of scaffold construction.

Among the 38 cases, 26 cases were significantly effective, 10 cases were effective, and 2 cases were general. The satisfaction rate of curative effect was 94.74%, sufficient to illustrate the effectiveness and innovation of our approach. Since the background wall of our early photo room was mixed in color, the collected photos would affect the visual effect before and after the operation. But this is a real shooting background, and we can’t do photo editing either. Although the background before and after surgery is somewhat different, it does not affect our data measurement of patients before and after surgery. In addition to our preoperative and postoperative analyses of nasal measurement indices, it was found that postoperative nasal length, height, and depth, labial angle and frontal angle were all larger than those before surgery, while postoperative nasal width was smaller than that before surgery. These results also demonstrate the statistical validity of the method.

In summary, when using the ear cartilage to construct the "twin towers" type of nasal stents, an extension of the caudal septum can effectively provide reliable columellar support, and at the same time, an increase in the tip can be accomplished with improved short nasal columellar retreat, the basis of the principle of "inertia," thereby increasing ear cartilage support and maintaining the long-term stability of rhinoplasty surgery. It is a safe and feasible method for nasal stent construction and nasal tip forming. Through careful preoperative evaluation and individualized surgical planning, this method is suitable for most rhinoplasty patients. For severe saddle noses, short contracture noses or small ear cartilage, it is recommended to remove the costal cartilage for nasal tip plasty.

## Conclusion

A "twin tower" folding ear cartilage nasal stent can effectively extend the caudal septum and provide reliable support for the nasal columella, increase the supporting force on the ear cartilage while increasing the tip of the nose, and maintain long-term stability after rhinoplasty. It is a safe and feasible method for nasal stent construction and nasal tip shaping.

## Supplementary Material

Supplemental Material
