# Supplementary material for: A new technique for Asian nasal tip shaping: "twin tower" folding ear cartilage transplantation
Source: Case Reports Plast Surg Hand Surg. 2022 Sep 14;9(1):207–13. doi: 10.1080/23320885.2022.2123807 (PMC9487980; doi:10.1080/23320885.2022.2123807)
Supplement: Supplemental Material [file ICRP_A_2123807_SM0256.zip › questionnaire a.docx]

Patient reported outcome measures (PROMs) – Aesthetic Part

1. Are you satisfied with the shape of your nasal tip?

o Yes o No Why? ______________________________

1. Are you satisfied with the straightness of your nose?

o Yes o No Why? ______________________________

1. Are you satisfied with the shape of your nose from the side?

o Yes o No Why? ______________________________

1. How well does your nose suit your face?

o Good o Bad Why? ______________________________

1. Are you satisfied with the overall symmetry of your nose?

o Yes o No Why? ______________________________

1. Are you satisfied with the auricular donor side?

o Yes o No Why? ______________________________
